# Distribution Features and Potential Effects of Serotonin in the Cerebrum of SOD1 G93A Transgenic Mice

**DOI:** 10.1523/ENEURO.0001-22.2022

**Published:** 2022-11-03

**Authors:** Pei He, Binjun He, Shu Li, Wen Chai, Wei Rao, Yu Zhu, Wenzhi Chen, Ping Zhang, Xiong Zhang, Haili Pan, Renshi Xu

**Affiliations:** 1 Department of Neurology, Jiangxi Provincial People’s Hospital, Clinical College of Nanchang Medical College, The First Affiliated Hospital of Nanchang Medical College, Affiliated People’s Hospital of Nanchang University, Nanchang, Jiangxi 330006, China; 2 Institute of Life Science, Nanchang University, Nanchang, Jiangxi 330031, China; 3 Department of Neurology, Maoming People’s Hospital, Maoming, Guangdong 525000, China

**Keywords:** 5-HT, amyotrophic lateral sclerosis, Mash1, neural stem cell, subventricular zone, tryptophan hydroxylase 2

## Abstract

Serotonin (5-HT) participates in the pathogenesis of amyotrophic lateral sclerosis (ALS), but its effects have not been completely clarified. Therefore, we observed the distribution features and potential effects of 5-HT in the cerebrum of G93A SOD1 transgenic (TG) and wild-type (WT) mice by fluorescence immunohistochemistry, Western blotting, ELISA, as well as motor function measurements. Both 5-HT and tryptophan hydroxylase-2 (TPH2) were mainly present in the limbic systems of the cerebrum, such as the glomerular layer of the olfactory bulb, nucleus accumbens, cingulate, fimbria of the hippocampus, mediodorsal thalamic nucleus, habenular nucleus, ventromedial hypothalamus nucleus, lateral hypothalamus area, dorsal raphe nucleus, and piriform cortex. TPH2 and 5-HT were expressed in cell bodies in the dorsal raphe nucleus and piriform cortex, while in other regions they were distributed as filaments and clump shapes in axons. The TPH2 distribution in the cerebrum of TG was significantly lower than that in WT in preset, onset, and progression stages. TPH2 expression in the fimbria of the hippocampus, mediodorsal thalamic nucleus, habenular nucleus, ventromedial hypothalamus nucleus and lateral hypothalamus area was increased in the onset stage and decreased in the progression stage, gradually decreased in the cingulate with disease progression and significantly decreased in the glomerular layer of the olfactory bulb and nucleus accumbens in the onset stage in TG. The number of mammalian achaete-scute homolog-1 in the subventricular zone (SVZ) in TG was significantly lower than that in WT, which was correlated with the TPH2 distribution. Double immunofluorescence staining showed that TPH2, mammalian achaete-scute homolog-1 and 5-HT were mainly expressed in neurons but rarely expressed in microglia or astrocytes in the piriform cortex. The relative fluorescence density of TPH2 in the cingulate region was negatively correlated with the disease severity. Our findings suggest that 5-HT plays a protective role in ALS, likely by regulating neural stem cells in the subventricular zone that might be involved in neuron development in the piriform cortex.

## Significance Statement

Serotonin (5-HT) transmitters have been identified to play some roles in the pathogenesis of amyotrophic lateral sclerosis (ALS), but their accurate effects are not completely known. Therefore, we further explored its possible roles in ALS pathogenesis. Here, we assessed the potential distribution features of 5-HT in different anatomic regions of the cerebrum of G93A SOD1 transgenic (TG) mice in different disease stages. We found that 5-HT and mammalian achaete-scute homolog-1 were significantly reduced in many anatomic regions of the cerebrum of ALS-like TG mice. Our data suggested that 5-HT could play a protective role in the pathogenesis of ALS, probably by modulating the proliferation of neural stem cells in the subventricular zone (SVZ), which might involve neurons in the piriform cortex.

## Introduction

Amyotrophic lateral sclerosis (ALS) is a degenerative disease of the nervous system that is caused by a progressive loss of motor neurons in both the brain and spinal cord. The worldwide prevalence of ALS is ∼0.17 per 100,000 people (Chiò et al., 2013; Hardiman et al., 2017), and the peak age of onset is ∼55 years old. The main pathology of ALS is characterized by progressive and selective damage to upper and lower motor neurons, such as progressively worsened pyramidal tract signs, bulbar palsy, skeletal muscle weakness, atrophy, and fascicular fibrillation. The progressive and selective damage to upper and lower motor neurons leads to the gradual atrophy and paralysis of the throat and limb muscles and even spreads to all muscles throughout the body. Diagnosis of ALS is currently mainly based on clinical signs and symptoms and neurophysiological examinations. There is no specific diagnostic biological biomarker. The prognosis of ALS is very poor. To date, targeted drugs capable of effectively reversing or controlling the onset and progression of ALS are lacking.

At present, the pathogenesis of ALS is unclear. It might involve human immunodeficiency virus or prion virus infection, metal element poisoning or a deficiency of certain trace elements, chromosome 9 open reading frame 72 (C9orf72), TAR DNA binding protein (TDP-43), copper/zinc superoxide dismutase 1 (SOD-1) and abnormal deposition of RNA-binding protein FUS (FUS) protein (Chiò et al., 2014; Li et al., 2016; Lu et al., 2016), oxidative stress, abnormal RNA metabolism (van Blitterswijk and Landers, 2010), axonal transport defects (Ferraiuolo et al., 2011), nonmotor neuron lesions and abnormal mitochondrial dysfunction.

Current information shows that ALS not only involves the damage and loss of motor neurons but also exhibits a close relationship with certain nonmotor neurons to cause secondary damage in the axons of motor neurons (Brockington et al., 2010). The present evidence indicates that in addition to the above-described pathogenic factors that cause motor neuron damage, many neural structures in the cerebrum are also involved in the pathogenesis of ALS. Based on the current view, ALS is a disease caused by multiple neural systems, including the motor and nonmotor systems. However, no consensus has been reached regarding participation of the nonmotor system in the pathogenesis of ALS. The damage to nonmotor neurons might result in a series of nonmotor symptoms, including gastrointestinal, vascular, autonomic, and neuropsychiatric dysfunction, such as the observed clinical and social cognition dysfunction, changes in executive ability, language, memory and behavior dysfunction, and abnormal neuropsychiatric symptoms, the ALS superimposed syndrome, depression, pain, and dyspnea, and the urinary incontinence, constipation, emotional instability, sleep disorders, salivation, dysphagia, blood lipids abnormalities, high blood pressure, and itching (Newsom-Davis et al., 1999; Witgert et al., 2010; Moreau et al., 2012; Taylor et al., 2013; McCluskey et al., 2014; Nübling et al., 2014; Chiò et al., 2017; Prado et al., 2017; Santangelo et al., 2017; Beeldman et al., 2018). Nonmotor symptoms cause great psychological and physical suffering in patients and severely diminish patients’ quality of life and survival time. Some evidence also demonstrates that the pathologic damage to motor regions spreads to the adjacent nonmotor area to produce nonmotor symptoms (Günther et al., 2016).

Most research has shown that specific neurons and glial cells in ALS contain phosphorylated TDP-43 (pDTP-43) inclusion bodies (Prasad et al., 2019). The pDTP-43 inclusion bodies spread from the initial focus to other brain regions via intercellular transmission (Cushman et al., 2010). Pathologic staging of pDTP-43 distinguishes ALS with inclusion bodies from ALS with noninclusion bodies based on the distribution pattern of pTDP-43 inclusion bodies in neurons, and the occurrence of motor symptoms is divided into four stages (Brettschneider et al., 2013). In the first stage, pDTP-43 lesions are located in the intracranial motor cortex, brainstem motor nuclei of cranial nerves V, VII, and X–XII, and spinal cord α-motor neurons. In the second stage, lesions spread from the frontal motor cortex to the prefrontal neocortex, pontine reticular node, and precerebellar nucleus in the form of direct and indirect afferent fibers (Van Deerlin et al., 2008; Suzuki et al., 2012). In the third stage, the pDTP-43 inclusion body further spreads from the frontal lobe to the back and orbit of the straight gyrus, neocortical area, temporal lobe, posterior central gyrus, and basal ganglia. In stage 4, lesions spread from the frontal lobe motor area to the anteromedial temporal lobe, hippocampal structure and other wider areas. However, there is currently a lack of sufficient evidence on the nonmotor symptoms of patients with ALS, and the underlying mechanism is still unclear. Based on pathologic staging, it can be assumed that the neural system lesions of ALS originate from the primary motor cortex and then project to lower motor neurons and subcortical structures through axons.

More than 50% of patients with ALS have neuropsychiatric symptoms. Serotonin (5-HT) is a monoamine neurotransmitter that affects happiness and mood (Young, 2007). A previous study proposed that 5-HT in the cerebrum might be related to ALS nonmotor symptoms. Approximately 300,000 5-HT-containing neurons are distributed in the cerebrum. The 5-HT neurons have a large number of branches, so the cerebral cortex is widely and densely innervated by the serotonergic neural system (Jacobs and Azmitia, 1992). According to the biological characteristics, chemical structure and pharmacological effects of 5-HT, 5-HT receptors are divided into seven families (5-HT_1–7_) and 14 subtypes (5-HT_1A/1B/1D/1E/1F_, 5-HT_2A/2B/2C_, 5-HT_3_, 5-HT_4_, 5-HT_5A/5B_, 5-HT_6_ and 5-HT_7_), which are widely distributed in the central nervous system (CNS) and play a very important role in the human body (Filip and Bader, 2009). Thus, in this wide neuron network, 5-HT can be considered as a main regulator of the CNS, and it plays a very important role in many physiological activities, such as consciousness, learning, memory, hormone secretion, the wake cycle, cardiovascular disease, respiratory function, the injury sensory system, immune system function, food intake, energy metabolism, thermoregulation, motor control, aggression, smell and related pathologic states (depression, anxiety, mania, etc.). Additionally, it plays a very important role in the etiology of schizophrenia, autism, obesity, drug abuse, migraine, and hypertension (Ciranna, 2006; Green, 2006; Alenina et al., 2009). The 5-HT system has a wide range of effects on target neurons and interacts with many other neurotransmitters, such as dopamine (DA), GABA, norepinephrine, glutamate, and various peptides, to control the excitability threshold of motor neurons. 5-HT also plays an important role in the outward development of neurites in the embryonic nerve body, the differentiation of neurons, and the growth of synapses. In patients with ALS, motor and nonmotor symptoms are attributed to dysfunction of these neurotransmitter systems (Vermeiren et al., 2018).

5-HT has many effects in patients with ALS. Glutamate excitotoxicity is one of the causes of ALS, and denervation of 5-HT neurons causes the loss of inhibition of glutamate release, leading to neurotoxicity of superior and/or inferior motor neurons (Ciranna, 2006). The 5-HT system has neuroprotective and anti-inflammatory effects in patients with ALS. Evidence has confirmed that the platelet 5-HT level is significantly reduced in ALS patients. The platelet 5-HT level is not related to the duration of disease, but it is positively correlated with the patient’s survival rate. Studies in animal models of ALS support that 5-HT may directly regulate onset and survival. 5-HT participates in a series of dysfunctions that are altered by ALS, including disorders of motor neuron excitability and energy metabolism (Dupuis et al., 2010).

Studies in ALS patients and transgenic (TG) ALS mice have shown an increase in energy expenditure during the course of disease (Desport et al., 2001; Funalot et al., 2009), which is similar to the chronic depletion effect of brain-derived 5-HT (Yadav et al., 2009). Some patients with ALS have difficulty swallowing, and the brainstem motor nuclei, such as the trigeminal nerve, facial nucleus, vagus nucleus, and hypoglossal nucleus, control swallowing and receive extensive 5-HT innervation from the brainstem and many other areas of the brain. Therefore, early supplementation with 5-HT can alleviate or delay the symptoms of dysphagia in neurologic diseases, provide patients with a better quality of life and reduce the risk of death for patients (Haney et al., 2019). 5-HT and norepinephrine increase the excitability of motor neurons, and DA, 5-HT, and noradrenaline (NA) neurotransmitters have been shown to play a major role in initiating and promoting the expression of spinal cord motor output, thereby controlling the spinal segmental reflex (Milan et al., 2014).

There is a relationship between serotonergic neurons and many primary respiratory nuclei that are essential for respiratory rhythms. Correlatively, it has been shown that the 5-HT neurons in the raphe nucleus play a spontaneous role in respiratory rhythm, and their firing frequency increases during inhalation, which is the key pacemaker of respiratory activity *in vitro* (Ptak et al., 2009). Serotonergic nerves may play a major role in respiratory rhythm and chemical sensitivity through the interaction between metabolites and the brainstem respiratory network (Hilaire et al., 2010; Hodges and Richerson, 2010). These findings indicate that the serotonergic system is a potential therapeutic target for the treatment of respiratory failure in patients with ALS.

Serotonin-related substances also play very important roles in ALS; for example, 5-HT and ATP promote the migration of microglia, which are the resident mononuclear phagocytes in the CNS and are related to the pathogenesis of neurodegenerative diseases such as ALS. In SOD1 G93A mice, microglial 5-HT_2B_ receptor expression is upregulated in the lumbar (L3–L5) spinal cord, and blocking the 5-HT_2B_ receptor on microglia can promote disease progression (Borkowski et al., 2020). The compensatory production of the HT_2B_ receptor may delay disease progression, and interfering with this receptor may be therapeutically useful.

Astrocytes are the most abundant glial cells supporting neurons in the CNS. The neuroprotective effect of astrocytes has been confirmed in various neurologic diseases, such as ALS, spinal cord injury, stroke and Parkinson’s disease (Gleichman and Carmichael, 2014; Nicaise et al., 2015; Miyazaki and Asanuma, 2016). Stimulating 5-HT1A receptors on astrocytes can promote the proliferation of astrocytes and upregulate antioxidant molecules (Miyazaki and Asanuma, 2016). Systemic injection of 5-HTP can delay muscle weakness and decrease muscle tone in the hind limbs of ALS mice. In addition, high-dose administration of the 5-HT precursor significantly prolongs the lifespan of ALS model mice. Administration of the 5-HT precursor in SOD1 G93A mice increases serum 5-HT, which can improve motor function and survival rates. These studies suggest that 5-HT metabolism plays an important role in SOD1 G93A mice (Gurney et al., 1994). As mentioned above, 5-HT plays a very important and active role in the pathogenesis of ALS, meriting explorations of the relationship between 5-HT and ALS. Consequently, we used SOD1 wild-type (WT) and G93A SOD1 TG mice to observe and analyze the distribution and altered characteristics of 5-HT in the different anatomic regions of the cerebrum in different disease stages, as well as the changes in both tryptophan hydroxylase-2 (TPH2)-positive and mammalian achaete-scute homolog-1 (Mash1)-positive cells in the subventricular zone (SVZ). The results showed that the 5-HT distribution was significantly decreased in the cerebrum of TG compared with WT mice. TPH2, Mash1, and 5-HT were mainly expressed in neurons but rarely expressed in astrocytes or microglia in the piriform cortex (Pir), while they were not overlapping in the other cerebral regions, implying that the neurons in Pir might be involved in the role of 5-HT in ALS. Our study further showed that 5-HT was a potential factor in the pathogenesis of ALS. The distribution of both 5-HT and Mash1 exhibited synchronized alterations in SVZ of both WT and TG mice, which implied that 5-HT might have a relationship with neural stem cells in exerting an effect on the pathogenesis of ALS.

## Materials and Methods

### Animal model

The C57BL/6 SOD1 G93A transgenic mice (TG; The Jackson Laboratory) were reproduced by mating male TG mice with C57BL/6 SOD1 wild-type female mice (WT) at the Jiangxi Provincial Institute of Neurology, Jiangxi Provincial People’s Hospital and Affiliated People’s Hospital of Nanchang University. Genomic DNA isolated from mouse tail tissues was used to detect whether mice were positive for SOD1 G93A TG by PCR. The primers used for PCR were as follows: the forward primer for IL-2 was 5′-CTA GGC CAC AGA ATT GAA AGA TCT-3′, the reverse primer for IL 2 was 5′-GTA GGT GGA AAT TCT AGC ATC ATC C-3′, the forward primer for hmSOD1 G93A was 5′-CAT CAG CCC TAA TCC ATC TGA-3′, and the reverse primer for hmSOD1 G93A was 5′-CGC GAC TAA CAA TCA AAG TGA-3′. The amplification conditions were as follows: degeneration at 94°C for 3 s, annealing at 60°C for 1 min and extension at 72°C for 1 min, for a total of 35 cycles. Mice were killed after anesthetization by intraperitoneal injection of 4 g/kg body weight chloral hydrate (100 mg/ml solution) at the designated time points in the preonset stage (60–70 d), onset stage (90–100 d), and progression stage (120–130 d; Rosen et al., 1993; Gurney et al., 1994; Henriques et al., 2010). In the different disease stages of mice, gastrocnemius muscles in abnormal limbs were subjected to hematoxylin-eosin staining biopsy, and the pathologic alterations of muscle were detected under a light microscope to observe the paralysis of limb muscle and further identify the preonset, onset, and progression stages of TG mice (Rosen et al., 1993; Gurney et al., 1994; Henriques et al., 2010; Zhou et al., 2015). The details about gastrocnemius muscle biopsy data of mice at the different time points and the reason for performing these assays can be found in our previously published paper (Zhou et al., 2015). The experimental protocols described above were the same as those in our published papers (Zhou et al., 2015; Liang et al., 2017). All animal studies and experiments were conducted in accordance with the Guide for the Care and Use of Laboratory Animals of China and were reviewed and approved by the ethics committee for animal care and use.

### Fluorescent immunohistochemical staining of the cerebrum

Both male WT and TG mice were anesthetized by intraperitoneal injection of chloral hydrate and perfused with 20 ml 0.9% saline and 40 ml 1× PBS pH 7.5 4% paraformaldehyde (PFA) at room temperature (RT). The brain was stripped and placed in 4% PFA buffer overnight and then incubated in 1× PBS pH 7.5 20% sucrose, followed by embedding using OCT compound. For fluorescent immunohistochemical staining of the cerebrum, the brain was selected for successive coronal cutting on a Leica cryostat and collected on Superfrost Plus slides, and the thickness of the sections was 12 μm. The sections were permeabilized with 0.2% Triton X-100 and blocked with 1× PBS containing 10% goat serum after rehydration in pH 7.4 1× PBS and incubated with the following primary antibodies: goat anti-serotonin (5-HT) antibody 1:200 (ab66047, Abcam Ltd.), rabbit anti-TPH2 antibody 1:400 (ab184505, Abcam Ltd.), rabbit anti-Mash1 antibody 1:100 (ab240385, Abcam Ltd.), mouse anti-TPH2 antibody 1:200 (ab211528, Abcam Ltd.), mouse anti-Mash1 antibody 1:200 (61271, Active motif Inc.), rat anti-serotonin antibody 1:200 (ab6336, Abcam Ltd.), rabbit anti-NeuN antibody 1:200 (ab177487, Cell Signaling), rabbit anti-GFAP antibody 1:200 (80788, Cell Signaling Technology Inc.), and rabbit anti-Iba1 antibody 1:200 (ab178846, Abcam Ltd.) at 4°C overnight, washed six times with 1× PBS 0.2% Triton X-100 for 5–10 min each time, and incubated with the following secondary antibodies: donkey anti-goat IgG H&L (TRITC): 1:200 (ab6882, Abcam Ltd.), donkey anti-rabbit IgG H&L (TRITC): 1:400 (ab6799, Abcam Ltd.), donkey anti-rabbit IgG H&L (TRITC): 1:100 (ab6799, Abcam Ltd.) conjugated to rhodamine (Red), or donkey anti-rabbit IgG H&L conjugated to Alexa Fluor 568, and goat anti-rat IgG H&L (TRITC) 1:100 (A11006, Life Tech Ltd.) or goat anti-mouse IgM/IgG H&L 1:100 (A10680, Life Tech Ltd.) conjugated to Alexa Fluor 488 for 2 h at RT and DAPI (Blue; ab228549, Abcam, Hong Kong Ltd.). The sections were then stained, extensively washed, mounted with antifade medium, observed and imaged under a Nikon E800 fluorescence microscope with a spot digital camera (Diagnostic Instruments) and Photoshop software (Adobe Systems). For double staining, nine sections from three mice were used.

### Analysis of positive expression

All positive cells of fluorescent immunohistochemical staining were assessed by counting the numbers or evaluating the relative fluorescence density (TPH2) of positive expression at 200× magnification in 10 sections per mouse and calculating the total number and relative fluorescence density of positive cells in all sections. The total number and relative fluorescence density were divided by the section number. Four to nine mice per group were used in this study, and the average number of positive cells was used for quantitative analysis. The number and relative fluorescence intensity of immunofluorescence staining were counted using Image-Pro Plus software version 6.0 (Media Cybernetics). As in our previous study (Zhu et al., 2020), the hanging wire test (Oliván et al., 2015) and ALS Therapy Development Institute (ALSTDI) score (from 1 to 5; Knippenberg et al., 2010) were used to assess the disease phenotype (motor dysfunction scale) of TG mice. In addition, the correlation between serotonergic neurons and disease phenotypes was analyzed.

### Western blotting

Western blot analysis was used to quantify the temporal changes in protein levels, and three mice per group were used. Briefly, brains were quickly removed from deeply anesthetized male WT and TG mice and stored at −80°C. After homogenization in lysis buffer containing protease inhibitor and phenylmethylsulfonyl fluoride, the protein concentrations of each group were assessed using BCA assays. The total protein was separated by SDS–PAGE (8%) with 3-mg protein in each lane and transferred to PVDF membranes. After blocking in 10% nonfat dry milk for 2 h at RT, the membranes were incubated with primary antibodies overnight at 4°C, followed by incubation with horseradish peroxidase-conjugated goat anti-rabbit or goat anti-mouse secondary antibody (1:5000, Santa Cruz Biotechnology) for 2 h at RT. Secondary antibody reactive bands were visualized with chemiluminescence reagents (ECL, Pierce) for 1 min and exposed onto X-films for 2–10 min. The band intensity was quantified using Image software and normalized to GAPDH internal controls.

### ELISA

Mouse ELISA kits for 5-HT were used to assess the temporal changes in 5-HT levels, and three mice per group were used. The brain tissues were homogenized in lysis buffer containing protease and phosphatase inhibitors (Sigma) and centrifuged at 12,500 × *g* for 10 min to obtain extract proteins. CSFs were collected from the cisterna magna. For each ELISA, 50 mg of protein or 5 ml of CSF was used. ELISA was conducted according to the manufacturer’s instructions (R&D Systems Inc., catalog #PM1700), and the standard curve was included in each experiment.

### Statistical analysis

Data in this study are expressed as the mean ± SD. Alterations in the concentrations of TPH2, Mash1 and 5-HT among groups, and the number and relative fluorescence density of positive expression in the same regions of the cerebrum in different disease stages and in different regions of the cerebrum in the same disease stages in both TG mice and WT mice, were compared by one-way ANOVA. Spearman’s coefficient was used for correlation analysis; *p* < 0.05 was considered statistically significant.

## Results

### Distribution features of 5-HT/TPH2 in different anatomic regions of the mouse cerebrum

**Figure 1.**
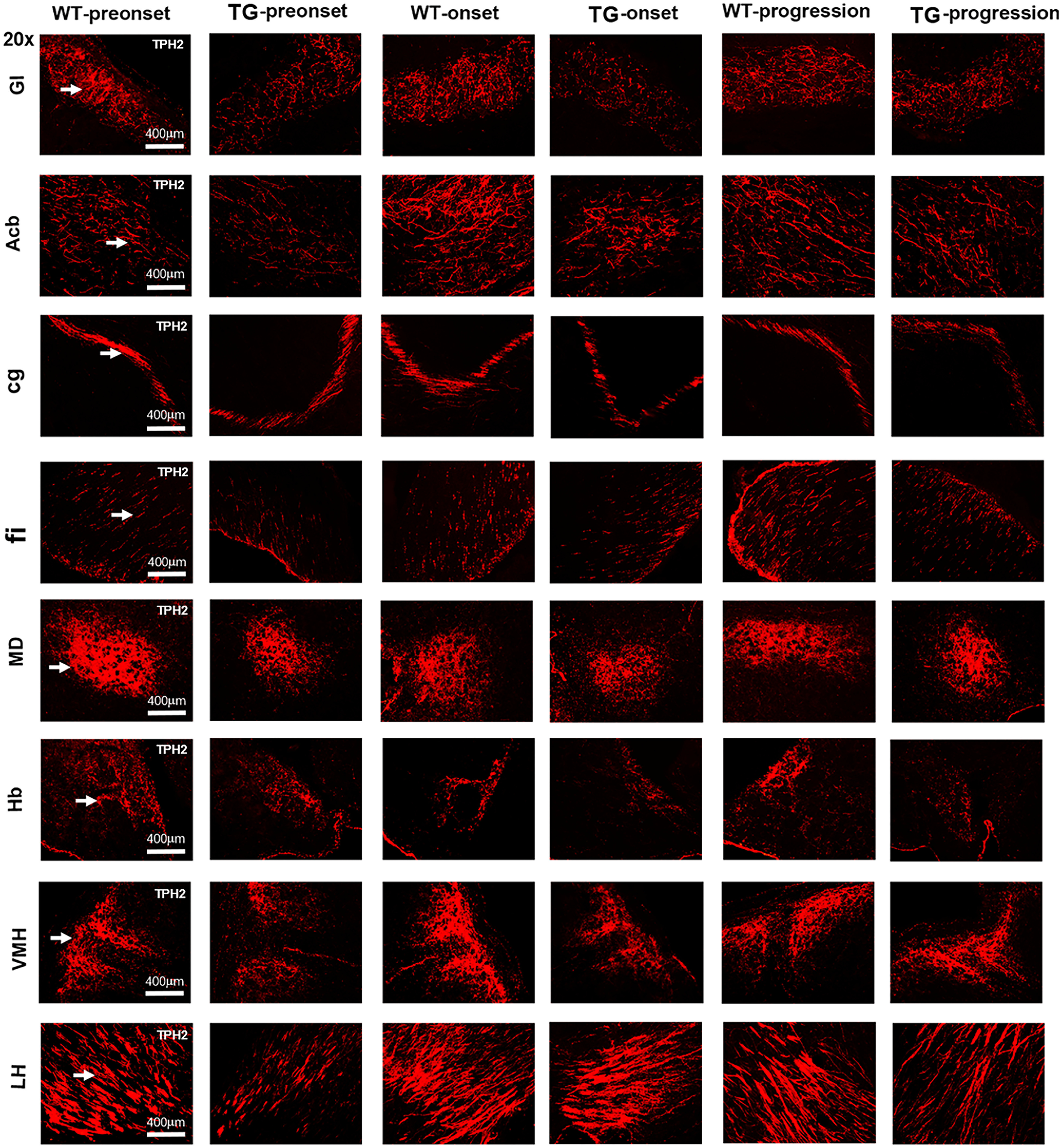
TPH2 distribution features in different anatomic regions of the cerebrum in different stages in TG mice and in the same periods in WT mice. Representative images showing the TPH2 distribution in the GI, Acb, cg, fi, MD, Hb, VMH, and LH regions of the cerebrum in different disease stages in TG mice and age-matched WT mice. *n* = 4–9 mice per group.

**Figure 2.**
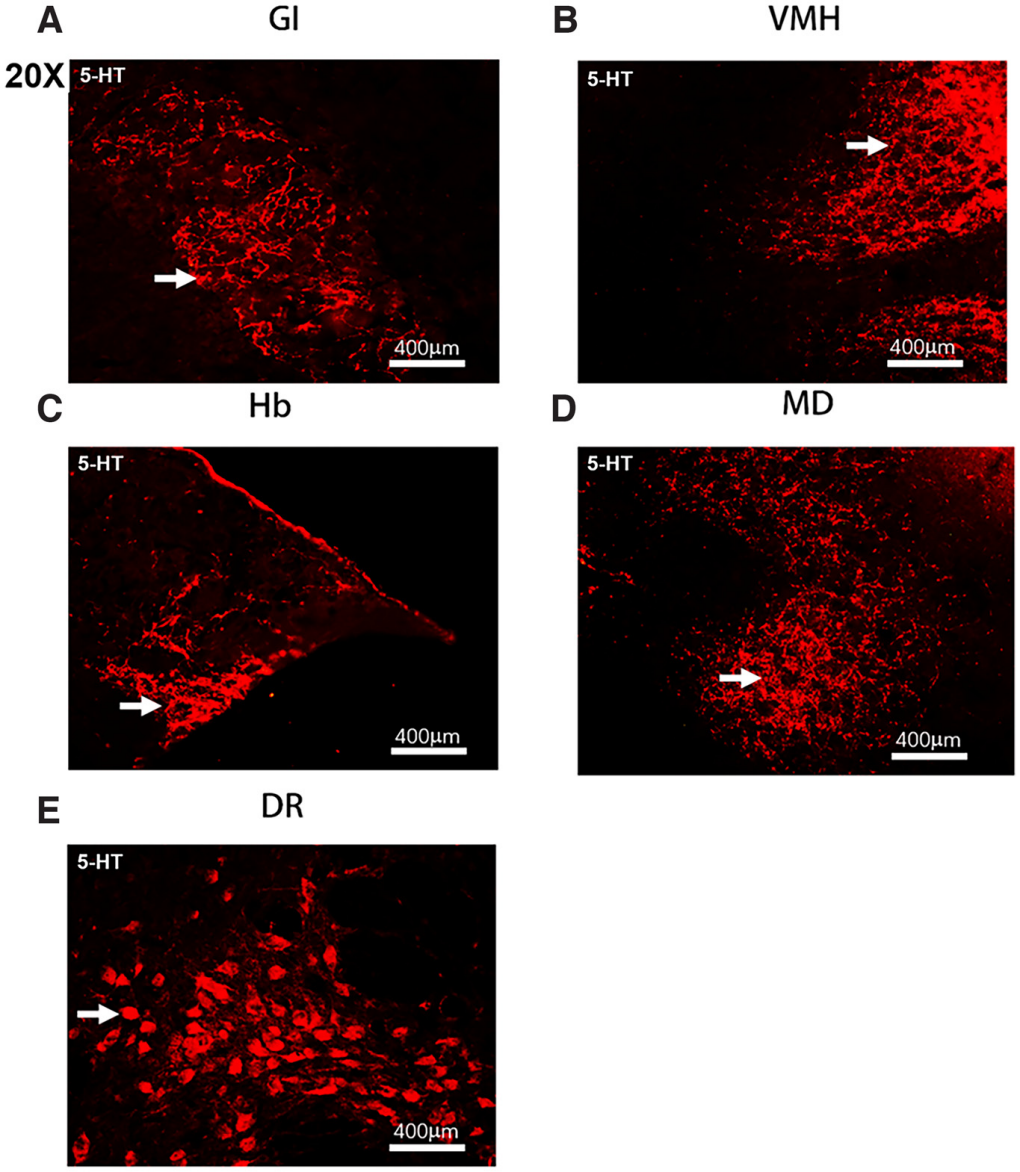
The 5-HT distribution pattern in the GI (***A***), VMH (***B***), Hb (***C***), MD (***D***), and DR (***E***) of the mouse cerebrum. 5-HT was distributed in the GI, VMH, MD, and Hb in filamentous and sheet-like shapes. 5-HT was distributed evenly in the neuron cytosol of cerebral nuclei such as DR.

To examine the potential effects of 5-HT in ALS, we observed its distribution features by fluorescence immunohistochemical staining with 5-HT and TPH2, a rate-limiting enzyme in the synthesis of 5-HT. In the mouse cerebrum, 5-HT and TPH2 had the same expression sites and morphologic characteristics, and they were mainly distributed in the limbic system and nonmotor anatomic regions of the cerebrum (Figs. 1, 2), such as the glomerular layer of the olfactory bulb (GI), nucleus accumbens (Acb), cingulate (cg), fimbria of the hippocampus (fi), mediodorsal thalamic nucleus (MD), habenular nucleus (Hb), ventromedial hypothalamus nucleus (VMH), lateral hypothalamus area (LH), dorsal raphe nucleus (DR), and piriform cortex (Pir). The most abundant expression of 5-HT/TPH2 was detected in the LH of the cerebrum, followed by the VMH and MD. 5-HT/TPH2 was distributed in Hb, VMH and MD nuclei in the form of nuclei and clumps, in LH and Acb nuclei by the axon form, in GI by filaments, in fi by a dotted line form, in MD under the third ventricle by a clump form, in cg by a rail bending shape, and in DR and Pir by a cell-like distribution (Figs. 1, 2).

### Comparison of the 5-HT/TPH2 distribution in three stages in age-matched TG and WT mice

**Figure 3.**
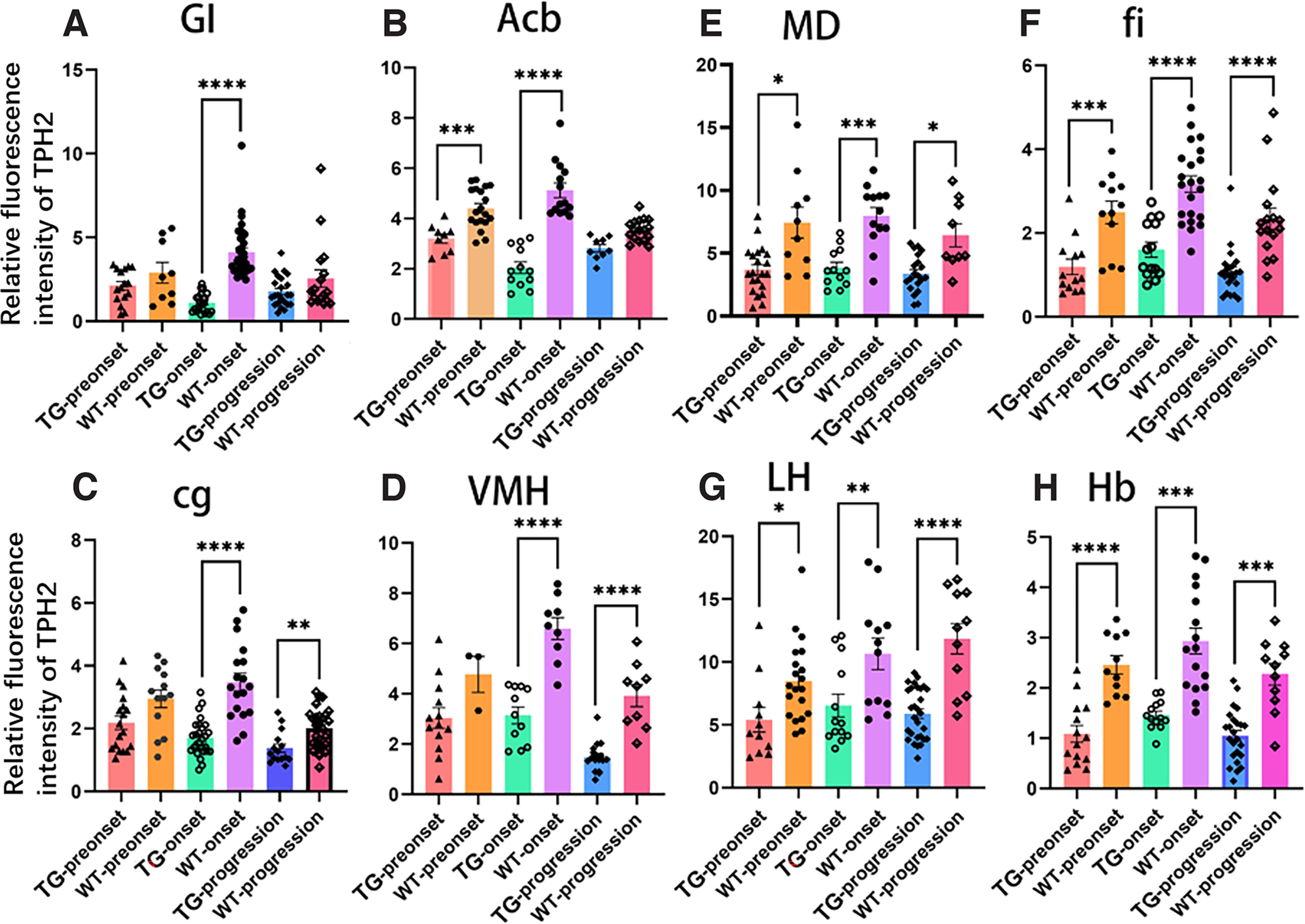
Quantitative analysis of the TPH2 distribution in the GI (***A***), Acb (***B***), cg (***C***), VMH (***D***), MD (***E***), fi (***F***), LH (***G***), and Hb (***H***) regions of the cerebrum between the different disease stages of TG mice and age-matched WT mice. *p <* *0.05, **p <* *0.01, ***p <* *0.001, ****p <* *0.0001. *n* = 4–9 mice per group.

Considering the wide distribution of 5-HT/TPH2 in the limbic system and nonmotor anatomic regions of the cerebrum, alterations in 5-HT/TPH2 expression in TG mice were detected. It was shown that the 5-HT/TPH2 distribution of TG mice was significantly lower than that in WT mice in the same stage. Compared with WT mice, TPH2 expression in TG mice was significantly decreased in the cerebral anatomic sites of GI (****p <* *0.0001; Fig. 3*A*), Acb (****p <* *0.0001; Fig. 3*B*), cg (****p <* *0.0001; Fig. 3*C*), VMH (****p <* *0.0001; Fig. 3*D*), MD (***p <* *0.001; Fig. 3*E*), fi (****p <* *0.0001; Fig. 3*F*), LH (**p <* *0.01; Fig. 3*G*) and Hb (***p <* *0.001; Fig. 3*H*) in the onset stage. TPH2 expression was significantly downregulated in the cerebral anatomic sites of cg (**p <* *0.01; Fig. 3*C*), VMH (****p <* *0.0001; Fig. 3*D*), MD (*p <* *0.05; Fig. 3*E*), fi (****p <* *0.0001; Fig. 3*F*), LH (****p <* *0.0001; Fig. 3*G*), and Hb (***p <* *0.001; Fig. 3*H*) in TG compared with WT mice in the progression stage. TPH2 expression was significantly decreased in the cerebral anatomic sites of Acb (***p <* *0.001; Fig. 3*B*), MD (*p <* *0.05; Fig. 3*E*), fi (***p <* *0.001; Fig. 3*F*), LH (*p <* *0.05; Fig. 3*G*), and Hb (****p <* *0.0001; Fig. 3*H*) in TG mice compared with WT mice in the preonset stage. These results implied that 5-HT probably played an important role in ALS.

### Comparison of the 5-HT/TPH2 distribution in different periods and in three stages in WT mice and TG mice

**Figure 4.**
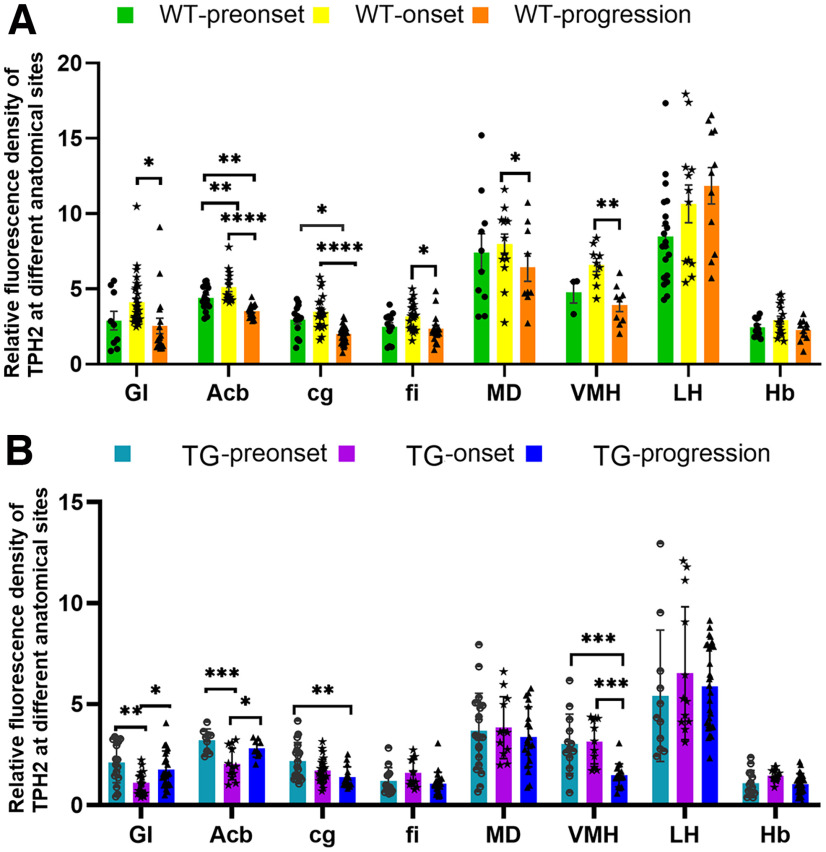
Quantitative analysis of the TPH2 distribution in different anatomic regions in different periods in WT mice (***A***) and different disease stages in TG mice (***B***). *p <* *0.05, **p <* *0.01, ***p <* *0.001, ****p <* *0.0001. *n* = 4–9 mice per group.

To analyze the potent relationship between 5-HT and the progression of ALS, the 5-HT/TPH2 distribution at different periods in WT mice and different stages of TG mice was observed. The 5-HT/TPH2 expression and distribution in the GI, Acb, cg, fi, MD, and VMH of WT mice were highest in the onset period and lowest in the progression period, which showed statistically significant differences (Fig. 4*A*). The 5-HT/TPH2 expression and distribution in the fi, MD, LH and Hb of TG mice was highest in the onset stage and lowest in the progression stage; however, there were no statistically significant differences. The expression of 5-HT/TPH2 in both GI and Acb was lowest in the onset stage, demonstrating a statistically significant difference, and 5-HT/TPH2 expression in both cg and VMH gradually decreased with the progression of disease from the preonset to the progression stage (Fig. 4*B*). Table 1 summarizes the changes in the TPH2 and Mash1 distribution in various anatomic regions of TG versus WT mice. These results indicated that 5-HT was closely related to disease progression.

**Table 1:** Comparison of TPH2 and MASH1 distribution in various anatomic regions between TG mice and WT mice

TG mice/WT mice				
Features	Regions	Preonset	Onset	Progression
TPH2	GI	-	↓	-
	Acb	↓	↓	-
	cg	-	↓	↓
	VMH	-	↓	↓
	MD	↓	↓	↓
	fi	↓	↓	↓
	LH	↓	↓	↓
	Hb	↓	↓	↓
MASH1	SVZ	↓	↓	↓

↓ denotes significantly decrease, - denotes no changes, / denotes comparison. GI, glomerular layer of olfactory bulb; Acb, accumbens nucleus; cg, cingulum; fi, fimbria of hippocampus; MD, mediodorsal thalamic nucleus; Hb, habenular nucleus; VMH, ventromedial hypothalamic nucleus; LH, lateral hypothalamic area; SVZ, subventricular zone.

### The distribution of Mash1-positive cells in the cerebrum of TG and WT mice

**Figure 5.**
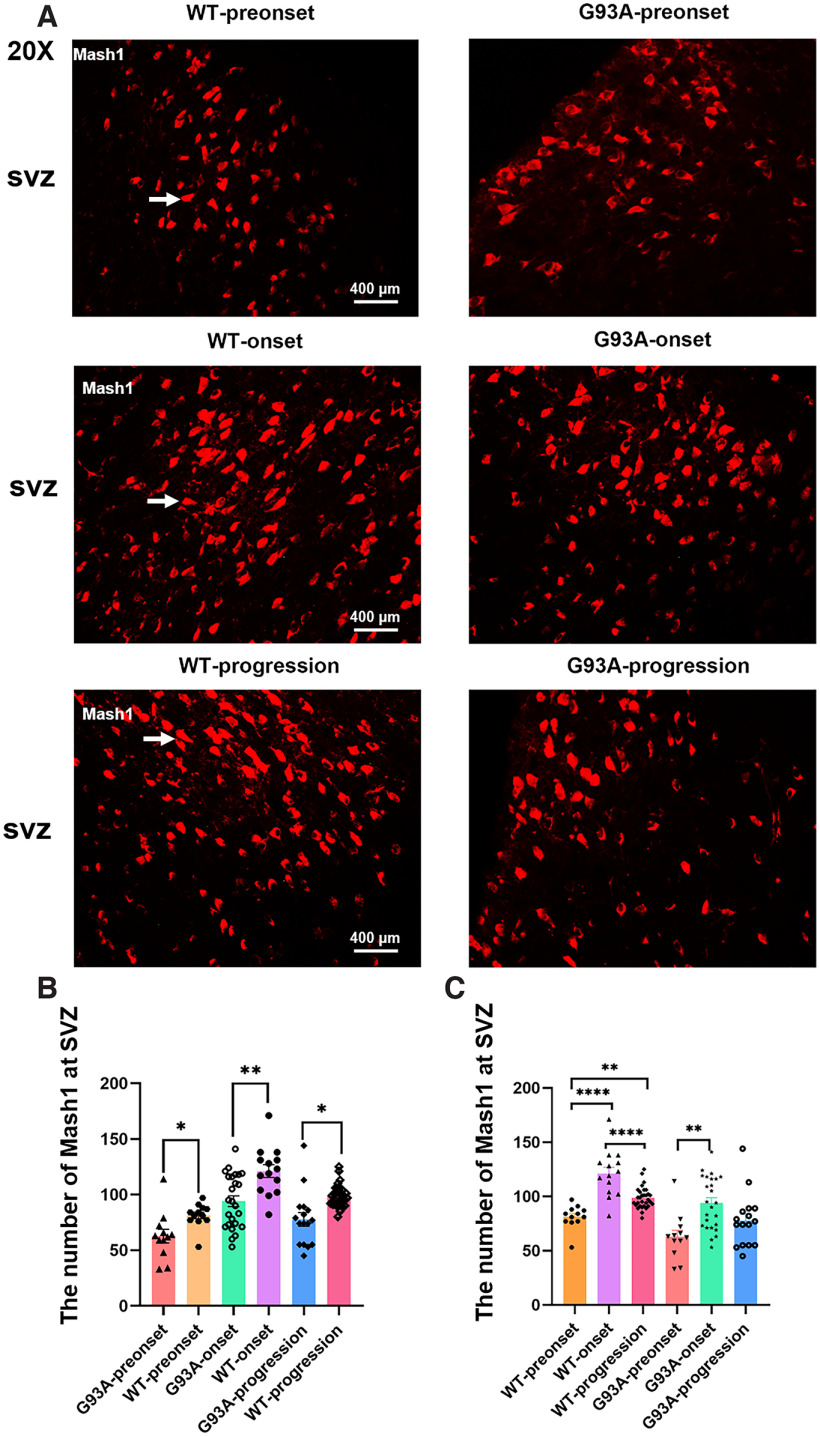
Mash1 distribution in the cerebrum in different stages in TG mice and the same periods in WT mice. ***A***, Typical immunofluorescence staining images of Mash1 in the subventricular zone (SVZ) of the cerebrum. ***B***, Comparison of the Mash1 distribution in the SVZ between TG and WT mice in different stages. ***C***, Comparison of the Mash1 distribution in the SVZ between different stages in TG and WT mice. *p <* *0.05, **p <* *0.01, ****p <* *0.0001. *n* = 4–8 mice per group.

Mash1 is a transcriptional activator that is necessary for 5-HT tryptaminergic neuron development. To further verify the potent role of 5-HT in the pathogenesis of ALS, Mash1 distribution alterations were observed in TG and WT mice. Mash1-positive cells were mainly distributed in the distribution region of neural stem cells in the cerebrum subventricular zone (SVZ) in the preonset, onset, and progression stages of TG mice and the age-matched same periods of WT mice (Fig. 5*A*). The number of Mash1-positive cells was significantly lower in TG mice than in WT mice in the preonset (*p <* *0.05), onset (**p <* *0.01), and progression (*p <* *0.05) stages (Fig. 5*B*). In WT mice, the number of Mash1-positive cells was significantly greater in the onset period than in the progression period (****p <* *0.0001), significantly greater in the progression period than in the preonset period (**p <* *0.01), and significantly greater in the onset period than in the preonset period (****p <* *0.0001; Fig. 5*C*). In TG mice, there were significantly more Mash1-positive cells in the onset stage than in the preonset stage (**p <* *0.01; Fig. 5*C*). The results showed that the number of Mash1-positive cells was significantly reduced in TG mice, which was similar to the changes in 5-HT/TPH2 in TG mice. From the preonset to the onset stage, Mash1-positive and TPH2-positive cells gradually increased, but they were significantly decreased up to the progression stage, indicating a potent role of 5-HT in the pathogenesis of ALS.

### Correlation analysis of the distribution of Mash1-positive cells in the SVZ and the 5-HT/TPH2 distribution in different anatomic regions of the mouse cerebrum

**Figure 6.**
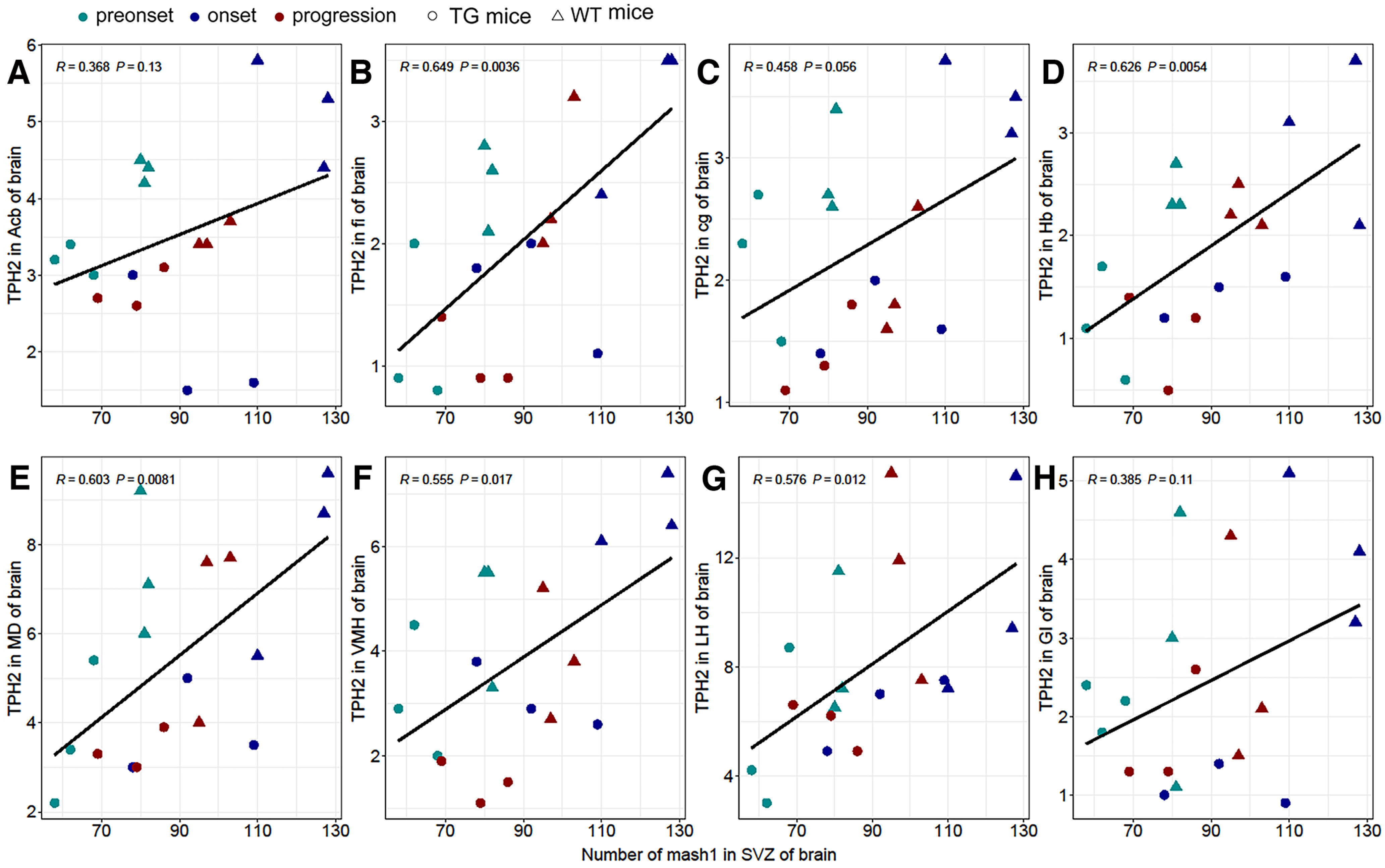
Correlation analysis of the Mash1 and TPH2 distribution in the SVZ of both TG and WT mice in different disease stages in Acb (***A***), fi (***B***), cg (***C***), Hb (***D***), MD (***E***), VMH (***F***), LH (***G***), and GI (***H***). *n* = 9 mice per group.

Correlation analysis of Mash1-positive cells in the SVZ and the 5-HT/TPH2 distribution in different distribution regions of the mouse cerebrum was performed to explore their strong correlation (Fig. 6). The results suggested that the change in Mash1-positive cells in the SVZ was significantly correlated with the change in TPH2 distribution in the different distribution regions of the mouse cerebrum (Fig. 6*B*,*D–G*), with *R* = 0.649 in fi (*p *=* *0.0036), *R* = 0.626 in Hb (*p *=* *0.0054), *R* = 0.603 in MD (*p *=* *0.0081), *R* = 0.555 in VMH (*p *=* *0.017), and *R* = 0.576 in LH (*p *=* *0.0012). The changes in Mash1 in the cerebrum were almost the same as those in 5-HT/TPH2 mice, with significantly lower levels of Mash1 in TG than in WT mice.

### Alterations in the expression of 5-HT/TPH2/Mash1 in different stages in the diencephalon of TG mice and age-matched WT mice

**Figure 7.**
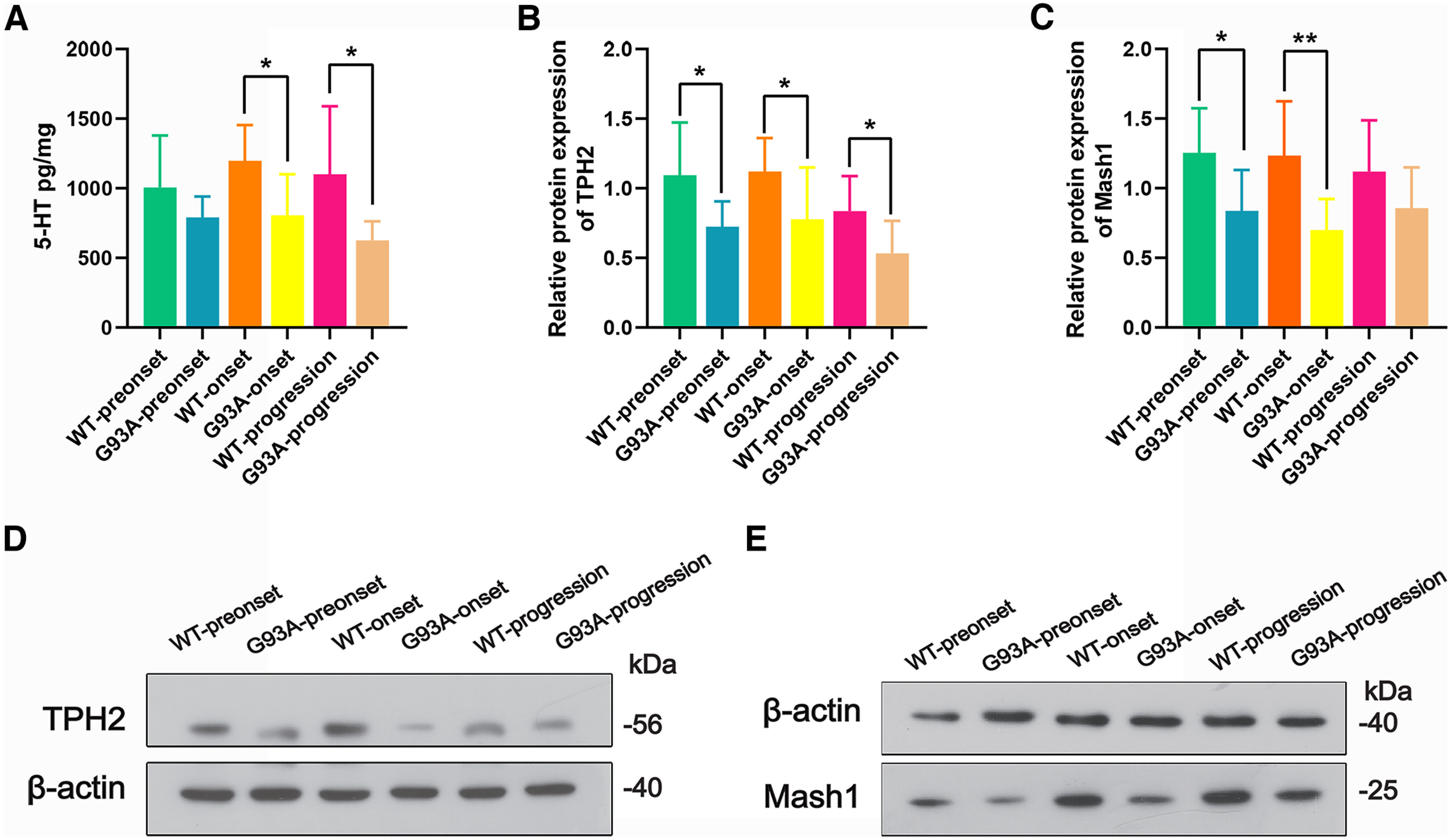
Alterations of 5-HT/TPH2/Mash1 levels in the diencephalon in different stages in TG mice and age-matched WT mice. ***A***, ELISA analysis showing 5-HT levels in the diencephalon at preonset, onset, and progression stages of TG mice and age-matched WT mice, *n* = 6 per group; Western blot analysis showing protein levels of TPH2 (***B***) and Mash1 (***C***) at preonset, onset, and progression stages in TG mice and age-matched WT mice. ***D***, ***E***, Representative Western blot bands of TPH2 and Mash1, respectively. *n* = 8 per group. *p < 0.05, **p < 0.01.

To further confirm the alterations of 5-HT/TPH2/Mash1 expression with disease progression, we examined the 5-HT levels and TPH2/Mash1 protein expression in different stages in TG mice and age-matched WT mice. ELISA results showed that the 5-HT level was significantly downregulated in the onset (*p <* *0.05) and progression (*p <* *0.05) stages of TG mice compared with age-matched WT mice (Fig. 7*A*). Western blot analyses showed that the relative protein expression of TPH2 was significantly lower in the preonset (*p <* *0.05), onset (*p <* *0.05), and progression (*p <* *0.05) stages of TG mice than in the age-matched WT mice (Fig. 7*B*,*D*). Mash1 protein expression was lower in the preonset (*p <* *0.05) and onset (**p <* *0.01) stages of TG mice than in the age-matched WT mice (Fig. 7*C*,*E*), confirming the expression alterations of 5-HT in TG mice with disease progression.

### The distribution of 5-HT/TPH2/Mash1-positive cells in the mouse cerebrum

**Figure 8.**
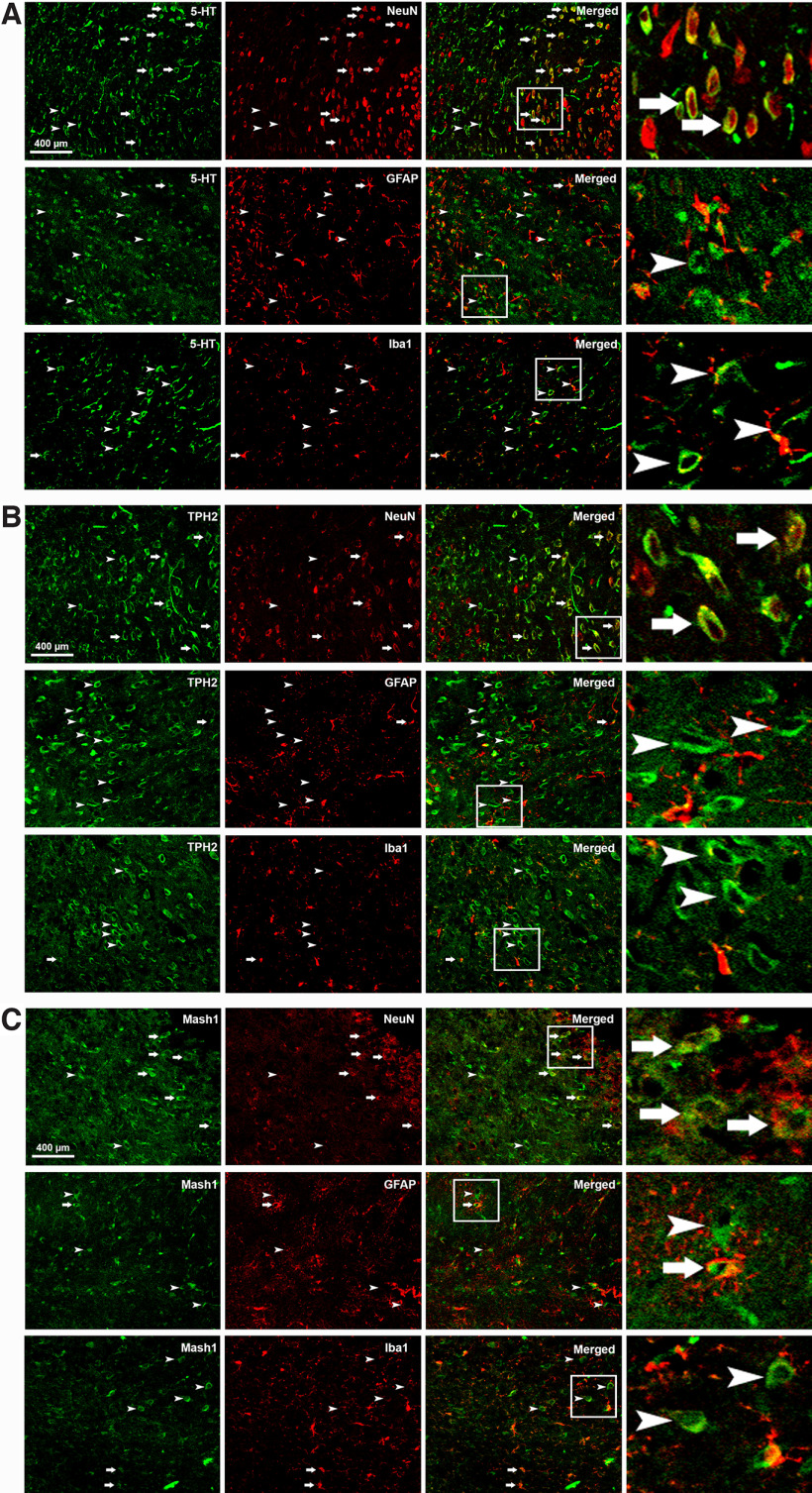
The 5-HT/TPH2/Mash1 distribution in neurons and glial cells in the Pir of mice. ***A***, Immunohistochemistry showing the proportion of 5-HT expression (green) in cerebral neurons (red, NeuN), astrocytes (red, GFAP), and microglia (red, Iba1). ***B***, Colocalization of TPH2 (green) with NeuN (red), GFAP (red), and Iba1 (red). ***C***, Double staining of Mash1 (green) with NeuN (red), GFAP (red), and Iba1 (red). Arrows indicate double-labeled cells. Arrowheads indicate cells expressing 5-HT, TPH2, or Mash1 but not NeuN, GFAP, or Iba1. The right panels show a higher magnification of the boxed areas. *n* = 9 sections from 3 mice.

To further clarify the localization of 5-HT/TPH2/Mash1 in the cerebrum, colocalization studies on 5-HT, TPH2, and Mash1 were conducted using the corresponding markers of neurons (NeuN), microglia (Iba1) and astrocytes (GFAP). Double immunofluorescence staining showed that 5-HT was mainly colocalized with NeuN (67.3%, *n* = 429 cells) but rarely colocalized with GFAP (1.5%, *n* = 914 cells) or Iba1 (1.4%, *n* = 478 cells; Fig. 8*A*) in Pir. Similarly, a large population of TPH2-positive cells coexpressed NeuN (70.6%, *n* = 967 cells) but rarely coexpressed GFAP (2.4%, *n* = 528) or Iba1 (2.3%, *n* = 656 cells; Fig. 8*B*) in Pir. Finally, Mash1-positive cells were found to largely overlap with NeuN (55.9%, *n* = 485 cells), while only a minor portion of Mash1-positive cells were intermingled with GFAP (3.1%, *n* = 464 cells) or Iba1 (4.9%, *n* = 473 cells; Fig. 8*C*) in Pir. However, there was no overlap of 5-HT/TPH2/Mash1 with NeuN/Iba1/GFAP in other cerebral regions (data not shown). These results indicated that the neurons in Pir might be involved in the effects of 5-HT in ALS.

### Correlation analysis of changes between the serotoninergic cell density and disease phenotype in TG mice

**Figure 9.**
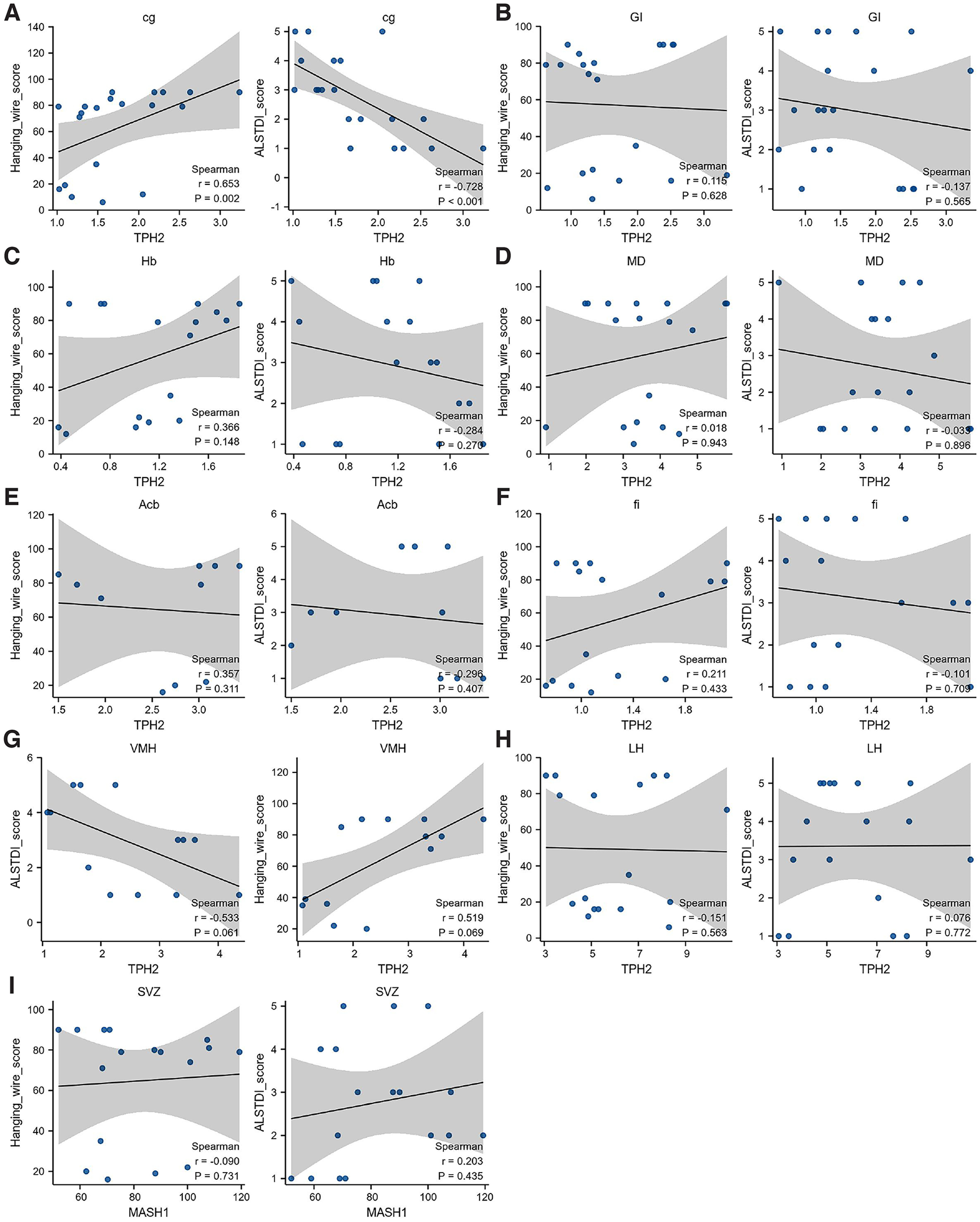
Correlation analysis of the change between the serotoninergic cell density and the disease phenotype in TG mice. ***A***, Correlation analysis of the density of TPH2 with the disease phenotype in cg. ***B–I***, Correlation analysis of the disease phenotype with the distributions of TPH2 in the GI (***B***), Hb (***C***), MD (***D***), Acb (***E***), fi (***F***), VMH (***G***), and LH (***H***) regions and the distribution of Mash1 in the SVZ (***I*)** region. *n* = 4–9 mice per group.

The above results strongly suggested that 5-HT played an important role in the pathogenesis of ALS. To further confirm this possibility, the correlation between changes in serotoninergic cell density and disease phenotype was analyzed. The correlation analysis showed that the density of TPH2-positive cells in cg (Fig. 9*A*) was significantly negatively correlated with the ALSTDI score (*R* = −0.728, *p *<* *0.001), while it was significantly positively correlated with the hanging wire score (*R* = 0.653, *p *=* *0.002). The distribution of TPH2-positive cells in the GI, fi, Hb, MD, Acb, fi, VMH, and LH regions did not correlate significantly with the disease phenotype (Fig. 9*B–H*). The distribution of Mash1-positive cells in the SVZ region did not correlate significantly with the disease phenotype (Fig. 9*I*).

## Discussion

ALS is a progressive neurodegenerative disease that is mainly involved in damaging motor neurons in the spinal cord, cerebral cortex and brainstem. Most patients die of respiratory muscle paralysis within three to five years after diagnosis, and no drug is currently available to treat or relieve this disease (Brylev et al., 2020). The exact pathogenesis of ALS has not been described to date. Most researchers focus on motor neuron lesions, while very few studies have examined nonmotor neurons. The nonmotor neuron lesions in ALS might have a close relationship with the pathogenesis of this disease. Thus, adequate attention should be given to nonmotor neuron lesions in ALS, which might directly or indirectly participate in disease development, for example, by directly causing nonmotor symptoms including neurosensory, psychiatric and autonomic symptoms, as well as sleep disorders, or by indirectly inducing motor neuron damage through nonmotor neuron lesions, such as DA, 5-HT, and NA neurons, as well as glial cells, increasing the activity of DA, 5-HT, and NA neurons to generate excitatory toxicity or activating glial cells to produce excessive inflammatory factors.

Almost all cerebral cortex areas exhibit the same distribution of the 5-HT system. 5-HT exists in the wide neuron network distribution in the cerebrum and can be regarded as one of the main regulators in the CNS. 5-HT plays a very important role in many physiological activities of the human body, such as depression, anxiety, mania, schizophrenia, autism, obesity, migraine, hypertension, food intake, energy metabolism, cardiovascular disease, respiratory function, thermoregulation, aggression, awareness, learning, memory, and smell. The clinical manifestations caused by 5-HT disorders are similar to the nonmotor symptoms of ALS. Fifty percent of ALS patients have neuropsychiatric symptoms, and 5-HT system disorders are related to the etiology and pathogenesis of various neuropsychiatric diseases. Therefore, we realized that 5-HT might be involved in ALS pathogenesis and innovatively conducted this study to address this idea.

In our study, the results showed that 5-HT/TPH2 was extensively distributed in the GI, Acb, fi, MD, Hb, VMH, LH, cg, DR, and Pir of the mouse cerebrum. Among them, the LH distribution in the cerebrum was the most abundant, followed by VMH and DM. Both TPH2 and 5-HT had the same distribution in the cerebrum, and their distribution patterns were also similar. Both TPH2 and 5-HT were distributed in different anatomic and functional regions of the mouse cerebrum in different shapes, mainly in the GI in a filament shape. In both cg and LH, they were distributed in an axon form. Both VMH and Hb were distributed between nuclei but not in the cell body. The distribution in fi was the same as that in both VMH and Hb distributed along the nucleus, showing a dotted line-like distribution. MD was distributed under the third ventricles in a clump-like distribution. None of the abovementioned distribution regions were distributed in cell bodies but were mainly distributed in the cytoplasm of 5-HT neurons in DR and Pir. In both cg and LH, especially in LH, a large amount of TPH2 was distributed via an axonal distribution. Perhaps the reduction in axons in the 5-HT system is also related to the pathogenesis of ALS. As expected, we found that the distribution of TPH2 in the cg region was positively correlated with the hanging wire score and negatively correlated with the ALSTDI score, which implied that the level of 5-HT synthesis in the cg region was negatively correlated with the severity of the disease in ALS mice. Additionally, considering the significant reduction in TPH2 density in the cg region in ALS, we speculated that the level of 5-HT synthesis in this region might have a neuroprotective role in ALS.

The 5-HT in the cerebrum is mainly derived from raphe nucleus neurons. Nine nuclei of 5-HT are divided into B1–B9. The axons produced by caudal nuclei (B1–B4) form the descending system, which mainly projects to the structure of the spinal cord, cerebellum, pons and midbrain. The cerebral cortex, hypothalamus, thalamus, basal ganglia, hippocampus and other structures receive more ascending fibers and links from medulla oblongata clusters (B5–B9; Jacobs and Azmitia, 1992). It has been found that ALS development might be caused by the gradual degeneration of neuron axons and ultimately lead to neuron death. Our results suggested that the axons labeled by 5-HT/TPH2 were significantly reduced. We observed the 5-HT/TPH2 distribution in the anatomic regions of the mouse cerebrum. These anatomic regions were not related to the motor system but were mainly in the nonmotor regions of the mouse cerebrum. We found that TG mice were more affected than WT mice. The 5-HT/TPH2 distribution was significantly reduced in TG mice in all stages. Our results indicated that 5-HT and 5-HTergic neurons were closely related to the occurrence of ALS and further showed that nonmotor neurons also participated in the pathogenesis of ALS.

It has been found that 5-HT levels and 5-hydroxyindole acetic acid (5-HIAA), the main metabolite of 5-HT, are reduced in the spinal cord of ALS patients after death, suggesting that 5-HT release is reduced. The level of tryptophan (the precursor of serotonin) in ALS patients is reduced, and the concentration of tryptophan in plasma is also reduced, with the lowest concentration found in ALS patients with the most severe scale (Sandyk, 2006). Brain-derived 5-HT levels are reduced in ALS patients and mice. During disease onset, the cell body of 5-HT neurons is significantly atrophied, accompanied by neurodegeneration (El Oussini et al., 2017). Reduced levels of 5-HT and its metabolites are found in brain tissue of ALS patients after death (Bertel et al., 1991), and increasing 5-HT transmission can maintain the excitability of motor neurons and compensate for the decline in motor function (Gurney et al., 1994).

The platelet 5-HT concentration in ALS patients significantly decreases, and the platelet 5-HT level is positively correlated with patients’ survival rate. The risk of patient death is reduced by 57% by 5-HT levels from 5-HT neurons and platelet-expressed serum in the normal range. Perinatal administration of the 5-HT reuptake inhibitor fluoxetine accelerates body weight loss and disease onset in ALS rats and increases the excitability of spinal motor neurons (Koschnitzky et al., 2014). Administration of 5-hydroxytryptophan (the precursor of serotonin) 5-HTP in TG mice increases the 5-HT content, thereby improving motor function and survival rates. Previous studies have shown that 5-HT levels are reduce in ALS patients, which is consistent with our results showing a significantly reduced 5-HT/TPH2 distribution in TG mice compared with WT mice in all disease stages.

Tryptophan hydroxylase (TPH) converts tryptophan C5 to 5-hydroxytryptophan (5-HTP). TPH is involved in the initial and rate-limiting steps in the biosynthesis of the 5-HT neurotransmitter. TPH is the rate-limiting enzyme in the synthesis of 5-HT. There are two tryptophan hydroxylase genes that encode two different homologous enzymes, TPH1 and TPH2 (71% sequence identity; Walther and Bader, 2003). TPH1 is mainly expressed in the skin, intestine, pineal gland and CNS, while TPH2 is only expressed in nerve cells, and its expression level is greater than that of TPH1. Consistent morphologic distribution characteristics of 5-HT and TPH2 are observed in the cerebrum. Therefore, we used TPH2 to replace 5-HT in the immunofluorescence staining to observe the distribution changes in 5-HT in the cerebrum.

To further verify the potential mechanism between 5-HT and the pathogenesis of ALS, we also studied and observed changes in the Mash1 distribution in TG and WT mice. Mash1 is a transcriptional activator that is necessary for the development of 5-HT tryptaminergic neurons and is widely expressed in the developmental stages of the central and peripheral nervous systems. Mash1 is expressed in neural precursor cells and acts as a coding transcription factor to regulate the differentiation of different neural progenitor cells (Guillemot and Joyner, 1993; Guillemot et al., 1993). It is extensively distributed in the spinal cord, the dorsal side of the neural tube, the dorsal midbrain and the ventral side of the telencephalon and hindbrain, prominently on the ventral side of the telencephalon, the ventral side of the diencephalon and the olfactory bulb epithelium. Reduced expression of the Mashl gene can significantly increase the differentiation of cortical progenitor cells into glial cells and significantly reduce the number of motor, sympathetic and parasympathetic nervous system, olfactory epithelial olfactory, and enteric neurons. Overexpression of Mash1 can promote embryonic and neural stem cells to differentiate into neurons. Mash1 and Nkx2-2 are coexpressed in the neuroepithelial region of the hindbrain to produce 5-HT neurons. Mash1 is essential for the production of 5-HT neurons, and it is also the determinant of the 5-HT phenotype (Chenn and Walsh, 2002; Hamada et al., 2006).

Our results showed that Mash1-positive cells were mainly distributed in neural stem cell regions of the SVZ, and the number of Mash1-positive cells was significantly reduced compared with that in WT mice, which is consistent with the significant reduction in 5-HT-positive cells in TG compared with WT mice. Western blot analysis confirmed the alteration of Mash1 levels in TG mice. Correlation analysis between Mash1-positive cells in SVZ and 5-HT/TPH2-positive cells of the mouse cerebrum showed a significant correlation in fi, Hb, MD, VMH, and LH. The Mash1 gene plays an important role in the process regulating the development and differentiation of the nervous system and is essential for the production of 5-HT neurons. The pathogenesis of ALS may be related to the expression level disorder of the Mash1 gene, which reduces the generation of 5-HT neurons, further causing a significant decrease in 5-HT in the CNS, which in turn directly or indirectly leads to ALS. In conclusion, our study suggested that the decrease in Mash1-positive and 5-HT-positive cells might be a pathogenic mechanism of ALS. 5-HT might serve as a palliative treatment to improve the quality of life and extend the survival of patients with ALS.

The projection path of 5-HT neurons in the cerebrum to both the brainstem and spinal cord should be further studied. Double antibody labeling of axons can be applied to identify those expressing TPH2 to further observe the relationship between the 5-HT axon and TPH2 distribution. 5-HT inhibitors or drugs that promote or inhibit 5-HT release can be applied to observe whether the lifespan of ALS mice is prolonged and the symptoms of muscle atrophy are aggravated or relieved, thus further confirming the protective effect of 5-HT on ALS.

In conclusion, 5-HT was distributed in the GI, Acb, fi, MD, Hb, VMH, LH, cg, DR and Pir of the mouse cerebrum, mainly in nonmotor areas. It was significantly reduced in the cerebrum of TG mice and widely distributed in neuronal axons. Mash1 levels were significantly reduced in the SVZ of TG compared with WT mice and significantly correlated with change in the TPH2 distribution. These findings indicated that changes in 5-HT were closely related to the distribution of Mash1, which might participate in the pathogenesis of ALS, likely through regulating neural stem cells involved in Pir development.

## Synthesis

Reviewing Editor: Francisca Bronfman, Universidad Andres Bello Facultad de Medicina Decisions are customarily a result of the Reviewing Editor and the peer reviewers coming together and discussing their recommendations until a consensus is reached. When revisions are invited, a fact-based synthesis statement explaining their decision and outlining what is needed to prepare a revision will be listed below. The following reviewer(s) agreed to reveal their identity: Matthew Butchbach, Krishna Aluri.

Dear authors, reviewers have agreed that the revision you have performed has greatly improved over the initial submission. However, they have suggested that the quality of written English must be improved before publication. The authors should solicit the assistance of English-language editorial service.

Additionally, the abbreviations for the various anatomical regions described in the abstract should not be used. Please replace with full names of the brain regions.
